# Proline Metabolism in Cancer: Emerging Roles in Redox Homeostasis and Therapeutic Opportunities

**DOI:** 10.3390/cancers17193156

**Published:** 2025-09-28

**Authors:** Tyrell C. Rossman, Gunjan Purohit, Oseeyi I. Daudu, Donald F. Becker

**Affiliations:** Department of Biochemistry, Redox Biology Center, University of Nebraska, Lincoln, NE 68588, USA; trossman@huskers.unl.edu (T.C.R.); gpurohit2@unl.edu (G.P.); odaudu3@huskers.unl.edu (O.I.D.)

**Keywords:** proline, cancer, metabolism, therapeutics, signaling, ROS

## Abstract

Cancer is a complex disease characterized by uncontrolled proliferation and progression toward metastasis. The activation and maintenance of these processes demand increased energy production. Traditional cancer therapies often target broad energy-generating mechanisms to inhibit cancer growth. However, the field of oncology is increasingly shifting toward more potent, precise, and personalized treatment strategies. This shift has fueled interest in novel cancer metabolic pathway targets. This review focuses on proline metabolism, an amino acid pathway that has been observed to be altered across various cancer subtypes. Proline has emerged as one of the most consistently deregulated non-essential amino acids in cancer biology. Proline metabolism is intimately linked to mitochondria function and energy regulation through mechanisms such as reactive oxygen species, ATP production, and interactions with the tumor microenvironment. Due to its dichotomous nature, proline metabolism functions are highly context dependent, varying across cancer subtypes. While this provides a potential novel therapeutic target, it also presents unique challenges and knowledge gaps.

## Simple Summary

Cancer can cause uncontrolled cell growth and spread to distant locations within the body, which requires large amounts of energy. To acquire the needed energy, cancer cells alter signaling pathways and harvest nutrients from the surrounding environment. One pathway that has emerged to be rewired is proline metabolism, in which the amino acid proline is utilized for protein synthesis and is catabolized in the mitochondrion, driving ATP production and redox homeostasis involving the NAD^+^/NADH balance and reactive oxygen species. Cancer cells increase the efficiency of proline-dependent energy production by changing signaling pathways, coupling metabolic and redox cycles, and balancing collagen production and degradation. Because of the multiple roles of proline in cancer progression, targeting proline metabolism has gained strong interest as a novel approach for inhibiting cancer progression. This review summarizes the various aspects of proline metabolism in different types of cancer and discusses strategies for developing new cancer therapeutics.

## 1. Introduction

Cancer is a multifaceted disease that remains an elusive enigma. A significant portion of cancer’s complexity is rooted in the diverse and broad changes within the metabolic and redox pathways. The metabolic fluctuations widely observed among cancer subtypes arise from adaptations that support rapid proliferation and metastasis. These phenotypes are broadly conserved hallmarks of cancer, which are achieved through diverse and distinct cellular mechanisms. The metabolic heterogeneity of these mechanisms often limits the development and research of universally effective cancer therapeutic strategies [1]. Current cancer therapeutics generally focus on targeting shared mechanisms between stage and tissue types, all with the unified goal of undercutting tumor growth and development [2]. The broadly conserved mechanisms of antitumorigenic drugs include the activation of tumor suppressing genes and the suppression of oncogenes [3,4]. The use of these traditional therapeutic drugs has proven useful on a broad population scale. While wide target antitumorigenic drugs have been useful, the medical field is evolving toward more potent and personalized treatment plans. This evolution is a result of the growing effort to improve patient outcome while reducing unintended side effects of cancer treatments.

To achieve this result, there is growing interest in more selective drugs that target tumor metabolism [5,6]. A variety of cancer metabolic pathways are currently being explored for novel anticancer drug targets with some pathways emerging as high potential candidates. The non-essential amino acid (NEAA) metabolic pathway has been revealed as a key contributor to cancer progression [7]. This has resulted in detailed studies of the NEAA pathway’s role and ability to promote observed cancer phenotypes. Previous studies of NEAA pathways has demonstrated the ability of NEAA’s to contribute to cancer phenotypes by driving proliferation, sustaining protein synthesis, aiding energy generation, balancing the redox microenvironment, and maintaining nucleotide biosynthesis [8,9,10]. One NEAA that has been strongly correlated to these cellular mechanisms is proline [11]. Proline is synthesized de novo in the mitochondrion, an organelle known for having altered metabolism across multiple cancer types [12]. Proline is a unique NEAA in that it contains a cyclic five-membered ring. The ring structure of proline has important roles in protein structures such as collagen, a major component of the extracellular matrix [13]. Proline benefits tumor progression by supporting protein synthesis, extracellular matrix building, and energy needs. Proline levels are regulated through specific metabolic enzymes and secondary regulators which will be delineated in this review for the identification novel cancer therapeutic opportunities [11,14].

## 2. Review of Proline Metabolism

### 2.1. Proline Biosynthesis, Catabolism, and Transport

**Figure 1:**
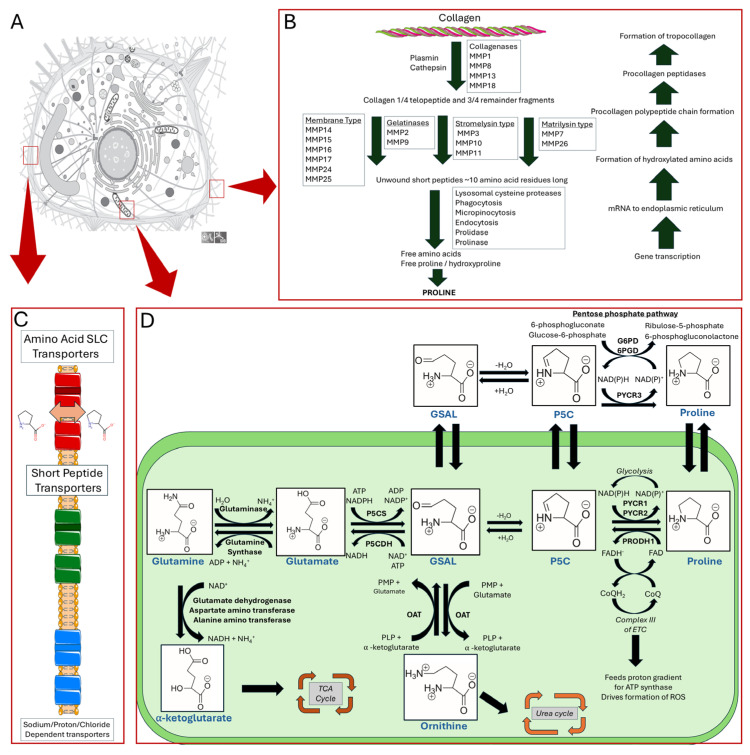
An overview of proline metabolism. Panel (**A**) illustrates a mammalian cell. Panel (**B**) details collagen formation and degradation pathways. Panel (**C**) highlights amino acid SLC transporters, short peptide transporters, and sodium proton/chloride-dependent transporters. Panel (**D**) shows proline biosynthesis and catabolic pathways and subcellular localization of enzymes in the mitochondria (depicted in green) and cytosol.

There are two primary sources of de novo proline biosynthesis: glutamine and ornithine. The metabolic pathways for both proline sources predominantly operate within the mitochondria, deriving L-glutamate-γ-semialdehyde (GSAL), the precursor for proline biosynthesis. Glutamine is converted to glutamate via glutaminase which liberates ammonia. L-Δ^1^-pyrroline-5-carboxylate (P5C) synthase (P5CS also known as ALDH18A1) is the bifunctional enzyme that next catalyzes the ATP-dependent conversion of L-glutamate into L-glutamyl-5-phosphate, with the concurrent production of ADP. The first reaction step is the phosphorylation of L-glutamate by the γ-glutamyl kinase domain to form the product, L-glutamyl-5-phosphate. This intermediate is then reduced to L-GSAL by the NADPH-reductase domain of P5CS, γ-glutamyl phosphate reductase. Ornithine, a component of the urea cycle, is converted into GSAL via ornithine-γ-aminotransferase (OAT), a mitochondrial pyridoxal-5-phosphate (PLP) dependent enzyme. GSAL undergoes spontaneous cyclization to form L-P5C. P5C and GSAL remain in equilibrium, with P5C undergoing hydrolysis to reform GSAL (Figure 1D).

The final step of proline biosynthesis is the reduction in P5C to proline (Figure 1). This reaction is catalyzed by P5C reductase (PYCR), utilizing either NADH or NADPH to form proline. Humans have three isoforms: PYCR1, PYCR2, and PYCR3 (also known as PYCRL). PYCR1 has been found to additionally convert piperideine-6-carboxylic acid to pipecolic acid [15]. The subcellular localizations of PYCRs are shown in Figure 1D. PYCR 1 and 2 are considered mitochondrial while PYCR3 is considered cytosolic [16]. The submitochondrial localizations of PYCR1 and PYCR2 are not yet established and remain an important question. It is known that PYCR1 and 2 can form a pentamer of dimers, forming a large decamer of approximately 350 kDa [17]. This leads to a likelihood that the PYCR decamer complex is the primary physiological form [18]. PYCR1 and 2 share a 97% active site similarity in sequence, whereas PYCR1 and PYCR3 share only 53% sequence identity [18].

Although the reactions catalyzed by the PYCR isoforms are redundant, they exhibit differences in specific activity and cofactor preference [16]. Mitochondrial enzymes PYCR1 and 2 exhibit higher specific activity than PYCR3 and show a strong preference for NADH, whereas PYCR3 prefers NADPH [16]. The subcellular localization of the enzymes are critical for their cellular function. For example, PYCR3 is critical for the biosynthesis of proline from ornithine in the cytosol [16]. PYCR1 and 2, are essential for proline biosynthesis in mitochondria. Amino acid biosynthesis is needed for cancer cell growth by supporting protein and nucleotide biosynthesis, and redox balance. Apart from this general role, PYCR3 generates NADP^+^ in the cytosol, which helps drive the oxidative phase of the pentose phosphate pathway (PPP) and thereby nucleotide biosynthesis (Figure 1D) [19,20]. Another important cellular function of PYCR1 and 2 is to support ATP production by supplying NAD^+^ for the TCA cycle [7,21].

The first step of proline catabolism is catalyzed by proline dehydrogenase (PRODH also known as proline oxidase, POX). PRODH oxidizes proline to P5C using flavin adenine dinucleotide (FAD) as the electron accepting coenzyme (reductive half-reaction). The reduced FAD produced through this reaction is re-oxidized via reduction of CoQ in the oxidative half-reaction catalyzed by PRODH. These reactions are carried out in the mitochondria where PRODH is localized to the inner mitochondrial membrane with access to CoQ of the respiratory chain [22].

There are two isoforms of human PRODH: PRODH1 and PRODH2. PRODH1 is selective for L-proline, whereas PRODH2 is selective for trans-4-hydroxy-L-proline, with PRODH1 forming P5C and PRODH2 forming L-Δ^1^-pyrroline-3-hydroxy-5-carboxylate [23]. P5C can spontaneously form the GSAL intermediate, which is then utilized by NAD^+^-dependent P5C dehydrogenase (P5CDH) to form glutamate and NADH. P5CDH is encoded by the ALDH4A1 gene and is more recently known as GSALDH [22]. Similarly, L-Δ^1^-pyrroline-3-hydroxy-5-carboxylate hydrolyzes non-enzymatically to the corresponding open-chain 4-hydroxy-glutamate- γ-semialdehyde, which is converted to 4-hydoxy-L-glutamate by GSALDH [23].

Proline obtained from the diet is absorbed as peptides in the digestive system. Most mammalian tissue studies on proline transport have focused on the epithelial barriers of the kidney and intestine, particularly in the context of nutrient absorption via the diet. When food is digested, proline-containing proteins in the stomach and small intestine are hydrolyzed by proteases and peptidases such as elastase, trypsin, aminopeptidase, pepsin, carboxypeptidases, and chymotrypsin [24,25]. The oligopeptides produced by these proteases are transported into enterocytes by gradient-driven peptide transporters such as peptide transporter 1 (SLC15A1 gene). Once absorbed across the intestinal system, peptides are broken down, leading to free amino acids. Peptides and free proline can be moved through different transporters, such as solute carrier family 6 member 19 (SLC6A19) and solute carrier family 6 member 7 (SLC6A7) [26,27]. Free proline can be transported via the H^+^ amino acid symporter PAT1 and the Na^+^ imino acid symporter SIT1. Basolateral transport of proline is believed to be facilitated by ATA2 and ASCT1 [28]. There are many proline transporters that can be categorized as either: amino acid SLC transporters, short peptide transporters, or sodium/proton/chloride dependent transporters, which are shown in Figure 1C. In addition to these cell membrane transporters, a sodium-dependent proline specific transporter is present in the mammalian brain [29]. Details on transporters specific for proline translocation across the mitochondrial membrane are scarce; however, a recent study found evidence for sideroflexin-1 in regulating proline transport in the mitochondrion [30]. The transporters for P5C are unknown. It remains unclear whether P5C crosses the mitochondrial membrane or if the open GSAL form is transported. The structure of the intermediate likely influences transport and the operation of the proline cycle.

### 2.2. General Proline Function

Proline is an important building block in protein structures. Proline’s unique cyclic structure often disrupts α-helices and facilitates the formation of turns in proteins [31]. In addition to being incorporated into proteins, proline as a free amino acid has a plethora of critical functions, such as combating ROS or assisting with regulation of osmotic pressure [32]. High proline levels are also known to help diminish protein aggregation caused by cellular stress [33]. Elevated proline levels in the cell can be achieved through the upregulation of proline biosynthesis or proline uptake from the environment.

Due to the positioning of proline residues at turn regions in proteins, they are accessible for post-translational modifications (PTMs), which can be used for the regulation and signaling of proteins [34,35]. This extensive regulation potential may offer significant advantages when considering cancer therapeutics. If an enzyme is sensitive to PTM-based regulation, there is an opportunity to identify analogs of the PTM molecule for the selective modulation of the enzyme. Although many proline analogs have been studied for their impact on proline metabolism, research on the PTMs of proline metabolic machinery remains limited [36].

The PTMs can induce domain and conformational shifts, enabling the broad regulation of a diverse range of enzymes [37]. An example of this form of regulation includes hydroxylation of HIF1α [38,39,40,41]. HIF1α has exposed proline residues, which under normoxic conditions, become hydroxylated, leading to its destabilization and the promotion of ubiquitination by Von Hippel–Lindau ubiquitin ligase. Once ubiquitinated, HIF1α will be degraded by proteasomes. HIF1α regulation by hydroxylation is just one example of many PTM pathways, which illustrates the broad regulatory potential of PTMs across the proteome.

Proline has additionally been linked to cell signaling [42,43]. Studies have delineated that high levels of cellular proline are associated with neurotoxic effects [44,45,46]. This increase in proline can impact synaptic functions that depend on gamma-aminobutyric acid (GABA) and glutamate signaling levels [47,48]. Proline shares structural similarity with GABA and has been shown to bind to GABA receptors (GAD), potentially reducing GABA levels and impairing central nervous system function [45]. Fluctuations in proline levels have led to recognition of proline as a signaling molecule. The regulation of proline signaling has been characterized as being regulated by the microenvironment, energy demand, and cellular stress conditions of the cell [49,50].

## 3. Proline Metabolism in Cancer

### 3.1. Proline Enzyme Expression Levels in Cancer

Proline metabolism exhibits diverse regulation at each enzymatic step across various cancer cell lines, making the metabolic pathway highly context-dependent. A list of each proline metabolic enzyme with their reported expression level and impact on cancer progression is provided in Table 1. The most evidence reported so far is for PRODH1 and PYCR1- the two key enzymes of the proline cycle. PRODH1 is frequently upregulated in cancer cell lines like OVCAR3, OVCAR5, MCF7, A549, HT29, PC3, and non-small cell lung cancer [19,51], but can be downregulated in some cases, such as in renal carcinoma 786-0 cells [52]. Recognized initially as a p53 inducible gene (PIG6), PRODH1 has an important role in driving apoptosis via proline oxidation and subsequent ROS generation through the ETC [53]. In this capacity, PRODH1 functions to limit cancer progression by promoting cell death through apoptosis. On the contrary, PRODH1 is also recognized as having a key role in cell survival, mediated by the proline cycle, which drives energy production via the ETC and coupling with the TCA cycle. Thus, PRODH1 upregulation has potential dual roles depending on cellular context, with either pro- or anti-effects on cancer progression. In cancers in which the proline cycle promotes metastatic growth, PRODH1 is squarely placed as a therapeutic target.

From Table 1, it is apparent that PYCR1 is commonly found to be upregulated in cancer cells and mostly promotes cancer progression. Increased PYCR1 expression has been observed in numerous cancer tissues and cell lines, including prostate cancer tissue samples, human B lymphoma cells, HCT116, OEC-M1, and H1299 cell lines [70,84,85,86,101,139,140]. Although, the majority of reports suggest that upregulation of PYCR1 promotes cancer progression, it has also been found to be downregulated in certain cancers such as in 138-treated pancreatic CAFs [97]. Similarly, other enzymes of proline biosynthesis have been reported to be upregulated and promoting cancer progression in certain cell types. For example, PYCR2 was found to be upregulated in glioblastoma multiforme patients and upregulated in LN229, SW1783, and HT29 cells lines, suggesting a role in cancer progression. PYCR3 also shows increased expression in cell types BT549 and PC9 [101,103,105,106]. PYCR1, PYCR2, and PYCR3 have been identified to have 12, 4, and 3 splice variants, respectively. The spliced forms of the proteins are most likely inactive, but could have regulatory functions through protein–protein interactions via their C-terminus [18]. These variants may contribute to the inconsistent role of the proline metabolic enzyme in different cancer conditions and warrant further investigation into the role of splice variants.

### 3.2. Cell Line Requirements for Exogenous Proline

Cell lines also show differences in proline metabolic enzyme expression that result in varied requirements for exogenous proline in the culture media. Cell lines with a strong dependence on exogenous proline include OVCA-420, PANC.04.03, CFPAC1, CALU-3, BxPC3, PANC.02.03, NCI-H441, HUP-T3, SU.86.86, and PANC.03.27. These cell lines typically exhibit lower proline biosynthesis and protein levels of P5CS, PYCR3, 4EBP1, and the ER chaperone. Proline independent cell lines such as SUIT-2, MIA-PACA-2, PATU-8988T, NCI-H23, Hs766T, A-375, OAW42, SK-MEL-2, NCI-H446, PK-59, and PATU-8902 synthesize sufficient proline levels in proline deprived media. Accordingly, these cell lines show increased P5CS and PYCR3 expression, Akt phosphorylation, and increased cap-independent translation. Blocking proline biosynthesis by knockdown of PYCR3 inhibits colony formation under proline-starving conditions demonstrating proline-dependent tumor cell growth [106].

In another study of human non-small cell lung adenocarcinoma PC9, cells cultured in DMEM media depleted of proline, the biosynthesis of proline was shown to be crucial for cell growth [19]. These findings indicate intrinsic differences in the proline biosynthesis capabilities of different cancer cell lines, which in turn determine their responses to changes in proline metabolic enzymes or metabolite supplementation. Further research is needed to understand how the expression of proline metabolic enzymes is regulated at the genomic level and how it contributes to cell-line specific variability.

### 3.3. Regulation of Proline Metabolic Enzyme Expression

To sustain rapid proliferation and development toward metastasis, cancer cells alter metabolic pathways to fulfill their energetic needs. The dynamic metabolic interplay between proline and cellular energy production appears to be positively correlated. This metabolic reprograming manifests distinctly across tumor cell subpopulations. As each cancer subpopulation has preferred metabolic requirements, the proline metabolic pathway is accordingly altered to assist in meeting cellular energy requirements. The two common points of regulation are the proline degradation and synthetic enzymes, PRODH1 and PYCR1, respectively.

PRODH1, integral to the proline-P5C cycle, has been extensively studied, with a ranging spectrum of reported results. These studies have identified that cancer signals have a clear and significant impact on the regulation of proline metabolism. Cancer cells regulate PRODH1 using oncogenes in response to hypoxic conditions and exogenous proline availability. Oncogene PPARγ ligand troglitazone induces PRODH expression in colon cancer cells (HCT116) through binding to the PPAR-responsive element at the PRODH promoter as well as by increased expression of the well-known regulator of PRODH expression, i.e., p53. Increased PRODH leads to an increase in ROS levels and thus induction of apoptosis [117]. Another important oncogenic transcription factor in proline metabolism is c-MYC, which is typically upregulated in many cancer subtypes. The overall effect of c-MYC is to increase proline levels through reciprocal regulation of PRODH1 and proline biosynthesis enzymes. c-MYC reduces PRODH1 levels transcriptionally and post-transcriptionally, whereas it induces the expression of P5CS and PYCR1. The PRODH1 promoter contains a canonical and a non-canonical c-MYC binding site, but chromatin immunoprecipitation (ChIP) assays showed that c-MYC does not bind directly to the PRODH promoter, suggesting regulation by c-MYC may be mediated through other transcription factors. The post-transcriptional regulation of PRODH levels by c-MYC is through upregulation of PRODH targeting microRNA- miR-23b [113].

Another important regulator of PRODH1 is AMPK, which is considered a contextual oncogene. A cellular model-based study designed to explore how cells adapt in matrix-deprived conditions (e.g., human fibroblast cells expressing SV40 LT antigen, ST antigen, hTERT, and h-RAS) found elevated AMPK activity (phosphorylation of AMPK at T172 residue) along with higher levels of ATP and proline. The matrix-deprived cells also showed an increased levels of P5CS and PRODH mRNA, where AMPK activation has no impact on P5CS expression, but causes a change in PRODH expression. These experiments were performed to understand why matrix detachment is a crucial step in cancer cells dissemination to distant sites through metastasis. Since matrix attachment is essential for adherent and non-transformed cells, for migration cancer cells need to overcome this challenge [141].

The role of hypoxic and ROS conditions on PRODH1 expression and function have been examined. PRODH expression was found to be upregulated under hypoxia in various cancer cell lines HT-29, HCT-116, HCT-15, 786-0, MCF7, Hs-578-T, PC3, M14, A549, and IGROV1-0.05% O_2_, as well as in the hypoxic microenvironment of a human breast cancer mouse xenograft model [109]. Hypoxia enhances PRODH expression through the activation of AMPK, not by HIF1α or HIF2α, leading to induction of protective autophagy through ROS generation (not apoptosis). Similarly, low glucose conditions during hypoxia also increase PRODH expression, but here it supports the cells by ATP generation [109]. The effect of ROS on proline metabolism was reported in a study of low-density lipoprotein (LDL) and oxidized LDL (oxLDL) that is associated with arthrosclerosis and cancer. The authors found that oxLDL (mainly 7-ketocolesterol-a, a major component of oxLDL) can induce PRODH expression in various cancer cells (OVCAR3, OVCAR5, MCF7, A549, HT29, PC3 cells) through PPARγ. The upregulation of PRODH is a protective response against oxLDL treatment, which causes beclin-1 mediated apoptosis to minimize the cytotoxic effects of oxLDL on cancer cells [111].

Regulation of PYCR enzymes has been explored in a similar context to PRODH1 by examining oncogene expression, hypoxic conditions, and exogenous proline availability. As briefly mentioned above, c-MYC upregulates proline biosynthesis. Silencing c-MYC leads to the upregulation of PRODH, P5CDH, and GS, but decreases levels of PYCR1, P5CS, and GLS proteins [113]. c-MYC transcriptionally and translationally induces the expression of all proline biosynthesis enzymes (P5CS, PYCR1, PYCR2, and PYCR3) in human B-lymphoma cells and MCF7 breast cancer cells. Interestingly, inhibition of PI3K activity by the compound LY294002 and wortmannin inhibits the expression of c-MYC, thereby downregulating proline biosynthesis enzymes. Additionally, c-MYC also regulates the levels of the precursor amino acid “glutamine” through activating the genes required for glutamine uptake (ASCT2, SN2), as well as repressing the glutaminase targeting microRNAs—miR-23a and miR-23b [142]. c-MYC binds to the promoter region of PYCR2 and upregulates its expression in breast cancer. Enhanced expression of PYCR2 in breast cancer correlates with poor prognosis. Increased PYCR2 activates AKT signaling to promote invasion and metastasis of breast cancer [102]. Kaposi’s sarcoma-associated herpesvirus (KSHV) is oncogenic and expresses a glycolytic transmembrane protein -K1, which is a lytic gene. K1 binds to PYCR1 and PYCR2, activating PYCR2 activity and making PYCR2 insensitive to feedback inhibition. This interaction increases tumors in mice as well as growth in 3D spheres [93]. The knockdown of proline synthesis enzymes P5CS, PYCR1, PYCR2, and PYCR3 in various cancer cell lines (P439. PC9, MCF7, MDA-MB-231, M14, and PC3) inhibits cell growth, however this effect is linked to reduced ATP levels and not mediated by apoptosis or a cell cycle arrest. Knockdown of proline biosynthesis enzymes also decreases the levels of glycolysis and lactate secretion [19].

The impact of oncogenes and the redox environment on PRODH1 and proline biosynthetic enzymes have also been studied in 3D culture models. Cancer cells grown in a 3D culture show maximum increase in proline synthesis and secretion. Expression of PYCR1 and PYCR2 do not change in 3D cultures across multiple cell lines tested, but an increase in PRODH level was observed (except PANC1 and ASPC1 cells, where PRODH was not detectable). Depletion of PYCR1, PYCR2, or PYCR3 partially inhibits proline production, whereas knockdown of P5CS completely inhibits proline production. Interestingly, supplementation of exogenous proline to P5CS-depleted cells is sufficient to rescue cell proliferation in 3D cultures. Other features of these 3D cultured cells include (A) a general reduction in protein synthesis, (B) a lower use of proline in protein synthesis, (C) increased collagen VI expression, (D) accumulation of cells in G1 phase (slower proliferation), (E) downregulation of ATF4 targeted genes, and (F) decreased synthesis of de novo amino acid synthesis enzymes (ASNS and PSPH) [95]. Additionally, the Fendt group exploited MCF10A H-Ras V12 cells to study the differences in metabolism in 2D versus 3D cultures (spheroidal growth to mimic attachment-free tumor conditions). They found inhibition of PRODH by L-THFA decreases ATP levels, which may be generated through complex III activity. The inhibition of spheroidal growth by complex-III inhibition, but not by complex-I inhibition, suggests PRODH-mediated ATP production supports spheroidal growth. The study also showed increased expression of PRODH in 3D cultured MCF7, HCC70, MDA-MB-231, 4T1, and EMT6.5 cell lines. The authors went on to show that proline levels are low in metastatic cancer sites, suggesting an increased catabolism upon metastasis. L-THFA, a PRODH inhibitor, was used in vivo and showed no effect in the primary tumor, but a significant decrease in metastasis [126].

Besides PRODH1 and PYCR, P5CS expression levels are also important to consider in cancer, as shown in a study focused on mesenchymal progenitor cells (10T1/2) and human cancer cell lines (HCT116 and PANC-1). The authors demonstrated the mitochondria’s response to cellular stress and energy-demanding conditions, such as serum starvation, was to form two distinct mitochondrial populations. One mitochondria population focused on energy production, reordering the mitochondria to increase oxidative phosphorylation capacity. The reordered mitochondria were also observed to have highly ordered cristae. The other mitochondria population was focused on proline synthesis. Mitochondria achieve this by enriching filamentous P5CS and disrupting cristae formation, and this leads to lower activity. The altered cristae structure and absence of ATP synthase led to extremely low mitochondrial energy production [143].

### 3.4. Enzyme Regulation

Enzymatic regulation of proline metabolism is another important factor to consider in cancer research. PYCR isoforms are inhibited differently by proline, with feedback regulation occurring at different intracellular proline concentrations. For example, values of feedback inhibition constants (*K*_i_) for L-proline range from ~0.1 mM (PYCR2), 1–2 mM (PYCR1), and 8 mM (PYCR3) [16]. Why PYCR2 is most sensitive to L-proline feedback inhibition is unknown [144]. The weaker inhibition of PYCR1 and PYCR3 suggest they have primary roles in proline biosynthesis in the mitochondrion and cytosol, respectively. Proline biosynthesis enzymes have been reported to be sensitive to changes in ATP levels. A classic study from 1957 demonstrated that an increase in ATP can cause inhibition of PYCR activity. The results of this study suggest that when ATP levels are low, PYCR activity may be elevated, leading to increased proline levels. Conversely, increased ATP levels may diminish PYCR activity [145].

PYCR1 activity has also been reported to be regulated by acetylation. Acetylation of PYCR1 by the CREB binding protein (CBP) at Lys228 in PYCR1 affects its decamer form and reduces enzyme activity. SIRT3, which is a well-known mitochondrial protein deacetylase, deacetylates PYCR1 thereby increasing PYCR1 activity. Acetylation-mediated inhibition of PYCR1 activity inhibits cell proliferation in MCF7 cells, whereas SIRT3-mediated deacetylation induces proliferation [83].

PRODH1 is known to be sensitive to lactate [146]. Kinetic and structural studies of the bacterial PRODH enzyme from *Escherichia coli*, proline utilization A (PutA), show L-lactate is a competitive inhibitor with respect to proline [147,148]. Cancer cells rely on aerobic glycolysis to assist in rapid cell growth and metastasis, resulting in a high level of lactate. Elevated lactate levels have been reported to correlate with high proline levels, which would be consistent with lactate-inhibiting PRODH1.

Humans have two isoforms of P5CS. The dominant long form, which contains an additional valine and asparagine on the N-terminus, is found throughout the body with regularly observed function and regulation. The short isoform is primarily located in the gut and is sensitive to ornithine feedback inhibition [149]. Both the long and the short P5CS isoforms produce the substrate GSAL, which is used for proline biosynthesis. Beyond P5CS isoforms, P5CS has been found to form large quaternary structures. P5CS can exist in rod- and ring-like structures changing in response to oxidative stress in the mitochondria [150]. Losing P5CS can lead to defects of mitochondrial respiratory complex organization. With proline metabolism primarily occurring in the mitochondria, there appears to be a connection between the regulation of proline and the state, health, and morphology of the mitochondria.

## 4. Proline Metabolism, Redox Homeostasis, and Hypoxia

### 4.1. Proline Cycle and Cancer

**Figure 2:**
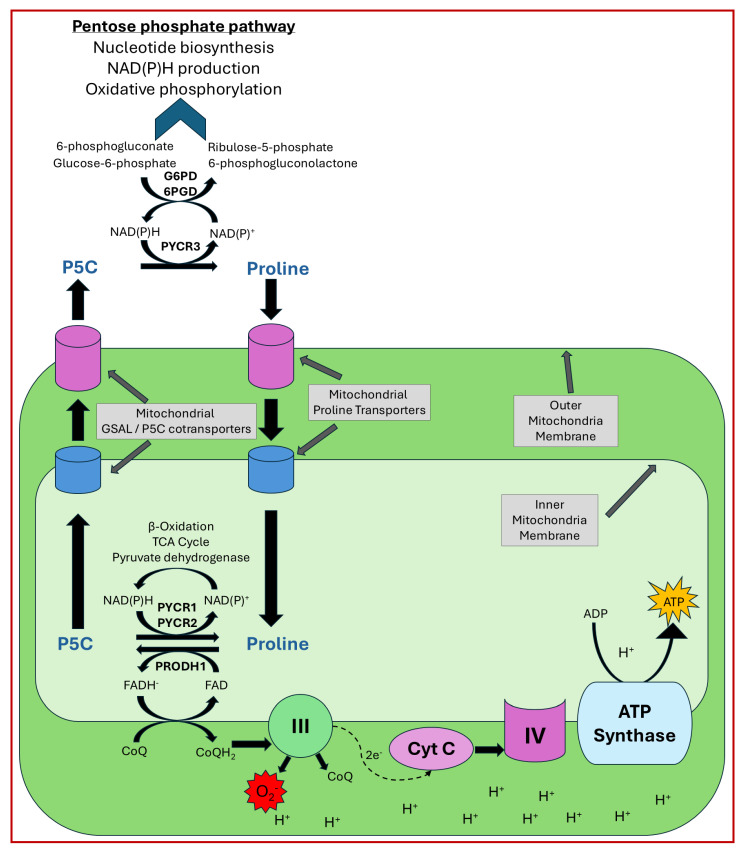
The proline cycle. Proline is proposed to cycle between the mitochondria and the cytosol. This cycling allows for the feeding of electrons via FADH and CoQH_2_ to the electron transport chain to assist in driving ATP production. The conversion of P5C to proline in the cytosol allows for the increased control of NADP^+^/NADPH levels thereby regulating the pentose phosphate cycle.

Research in the early 1970s identified P5C as both a biosynthetic precursor and a catabolic intermediary of proline metabolism. Based on the fact that the biosynthesis and degradation of proline involves reducing equivalents, Phang et al. proposed a model of the cyclic conversion of proline through its intermediate, P5C [151]. Similarly to the malate–aspartate shuttle, the function of the proline cycle was proposed to shuttle reducing equivalents to maintain mitochondrial respiration independently of the TCA cycle. Initial evidence came from Phang’s group, where they used radioisotope-labeled metabolites to confirm the transfer of reducing equivalents into the mitochondria upon interconversion of proline and P5C, as well as the transfer of electrons from these reducing equivalents to oxygen [152,153]. Supporting these findings, it was later shown that PRODH localizes in close proximity to the ETC complex and interacts with the CoQ1 subunit of complex III [154]. Phang et al. also showed that erythrocytes and hepatocytes can form an intercellular metabolic cycle in an in vitro setup, wherein hepatocytes catabolize proline and release P5C, which is then taken up by erythrocytes to convert back into proline. The resultant oxidation of NADPH in this process activates the pentose–phosphate pathway in the erythrocytes (Figure 2) [155]. These initial lines of evidence provided strong support for the proline cycle hypothesis, but in cellulo or in vivo evidence was completely lacking.

The first in cellulo evidence of the proline cycle came from a cancer cell line study from the Phang lab in 2015. They used P493, a lymphoma cell line, and PC9, a human non-small cell lung adenocarcinoma cell line, to demonstrate that the knockdown of PYCR1, 2, 3, or PRODH produces similar inhibitory effects on PC9 and P493 cell proliferation. Silencing the PYCRs also led to reduced glycolysis. Interestingly, supplementing knockdown cells with either P5C or proline did not rescue cell proliferation, suggesting that proline itself is not responsible for the growth defect; rather, the biosynthetic process is important for providing reducing equivalents. Indeed, the PYCR knockdown cells were found to contain lower levels of NAD^+^, NADP^+^, NADH, and NADPH, indicating suppression of respiratory chain activity and glycolysis. However, the authors were unable to provide a mechanism for glycolysis inhibition upon PYCR knockdown or conclusive evidence for intra-cellular compartmentalized cycling of P5C and proline [19].

Later, the Fendt group reported evidence of the proline cycle while studying differences in the metabolism of 2D versus 3D cultures. They found increased PRODH expression and proline consumption from media only in 3D growth conditions. Inhibition of PRODH by L-THFA or siRNA-mediated knockdown resulted in increased intracellular proline levels. Interestingly, the removal of proline from the media did not impair spheroid growth; instead, P5C recycled back to proline via proline-biosynthetic enzymes (mainly PYCR1) to refuel the PRODH substrate. The result of this proline cycle was a decreased NADPH/NADP^+^ ratio [126]. However, the authors did not address why PYCR3 knockdown, which forms the cytosolic arm of the proline cycle and preferentially utilizes NADPH, did not affect spheroid growth. It is possible that under certain conditions, the proline–P5C cycles within the mitochondria and connects to different metabolic pathways to oxidize NADPH in the cytosol, regardless of PYCR3 status. In another study, a similar increase in PRODH expression and proline levels was observed in the 3D culture of various cancer cell lines. To test the possibility that proline cycling causes proline accumulation, deuterated proline was provided to both 2D and 3D cells, but no difference was observed in the ratio of D_6_^−^ and D_7_-proline, indicating an unknown mechanism of proline accumulation [95].

A recent study reported the deubiquitinase ubiquitin-specific peptidase 9 X-linked (USP9x) as a positive regulator of PYCR3. Since PYCR3 controls the cytosolic arm of the proline cycle, deletion of either PYCR3 or USP9x affects the proline cycle-connected pentose phosphate pathway and shows a reduced NADP^+^/NADPH ratio [20]. However, this conclusion was primarily based on preliminary in vitro studies by Phang et al. and lacked specific testing of the role of the proline cycle’s functional role.

Despite all this evidence, the proline cycle model has yet to gain universal acceptance. Skepticism is partly due to findings that PYCR3 prefers P5C derived from ornithine, whereas PYCR1/2 prefers glutamine-derived P5C. Furthermore, PYCR3 preferentially uses NADPH, whereas PYCR1/2 use NADH for substrate reduction [16]. These differential preferences for substrates and cofactors suggest that, unlike its current simple model, the proline cycle may be a complex process that is context-dependent. Variation in the expression of proline metabolic enzymes across different cellular systems, as well as cell line-dependent differences in enzyme activity, may also contribute to the complexity of the cycle. Also, the submitochondrial location of proline metabolic enzymes is not well known, which is essential to understand how substrate transfer occurs between the proline cycle enzymes. The proline cycle may be restricted to a single mitochondrial compartment or extend to multiple compartments via interconnected metabolic pathways. Nonetheless, the available evidence cannot be overlooked, as the roles of the proline cycle in aerobic glycolysis, the pentose phosphate pathway, nucleotide biosynthesis, mitochondrial function, ROS generation, and cancer metabolism hold significant potential for cancer therapeutic development [19,126,153].

### 4.2. PRODH in ROS Signaling and Hypoxia

Hyper proliferative, biomass favoring, and energy-dependent cancer cells can rewire the ETC to promote rapid flux of carbon through the TCA cycle and oncogenic metabolic pathways. The TCA cycle feeds electrons to the ETC via FADH and NADH. With the TCA cycle being rapidly upregulated, the ETC can increase its flow creating a massive proton gradient which can lead to a dramatic increase of ROS. To counteract the high ROS levels, cells utilize uncoupling proteins (UCPs) to dissipate the proton gradient by leaking protons. This helps reduce protonic backpressure on the ETC, alleviating and preventing major oxidative distress [156].

PRODH activity in the mitochondrion can impact cell signaling pathways through different mediators such as ROS, ATP, COX2, HIF1α, and UCP2 [156]. PRODH drives ATP formation via the oxidation of proline coupled with electron transport through the ETC [157,158,159]. Generally, the downregulation of PRODH decreases ROS levels, whereas upregulation increases ROS as a byproduct of PRODH-catalyzed proline metabolism. Cancer cells upregulate PRODH levels to feed the ETC, generating ATP while also producing ROS [160]. In ZR75-30 breast cancer and DLD-1colorectal cancer cell lines, PRODH upregulation is essential for mitochondrial respiration and elevated H_2_O_2_ levels. PRODH upregulation was found to be particularly necessary under hypoxic or glucose starvation conditions [121,161]. PRODH regulators include oncogenic and tumor suppressor factors such as PPARγ, low density lipoproteins, p53, AMPK, and lactate [12,19,109,162].

ROS derived from PRODH activity can activate cellular responses like HIF1α to promote hyperproliferation and cell survival [13]. HIF1α is part of the HIF family of proteins that are stabilized by anaerobic conditions. Under normoxic conditions, proline residues in HIF1α can be hydroxylated by prolyl-4-hydroxylases -PHD1, PHD2, and PHD3, resulting in proteasomal degradation through ubiquitination by Von Hippel-Lindau ubiquitin ligase (VHL) [163]. Under hypoxic conditions, HIF1α is no longer hydroxylated and translocates to the nucleus associating with other HIF protein subunits to promote anerobic response proteins such as ANGPT, Nip3, and CTSD [164,165,166]. HIF1α stabilization under hypoxia also leads to the upregulation of genes like CA9 and GLUT1 and alters other pathways such as proline metabolism to assist cell survival [21]. Hypoxia also activates AMPK to counteract nutrient stress by inducing catabolism of nutrients leading to the increase in ATP production. This includes the activation of PRODH resulting in ROS production, stabilization of HIF1α, and increased glutamate generation [167]. Newly generated glutamate can be funneled into different energy metabolic pathways, such as the TCA cycle, to support cell growth. In this context, PRODH and the proline cycle are leveraged to feed glutamate into alternative aerobic respiration pathways [168].

Another cellular response to ROS is the activation of NRF2, a transcription factor encoded by the NFE2L2 gene, which is crucial for oxidative stress response signaling. In the inactive form, NRF2 is bound to KEAP1, a protein sensitive to oxidative stress. When cells undergo redox stress, ROS or other electrophiles react with cysteine residues in KEAP1 to promote the release of NRF2. Once released, NRF2 increases the expression of major detoxication enzymes, such as thioredoxins, peroxiredoxins, glutathione S-transferase alpha 2, and NADPH quinone oxidoreductase 1 to help mitigate ROS-induced stress [169]. The activation of NRF2 and HIF1a are cell survival mechanisms that potentially respond to elevated ROS due to PRODH activity and the proline cycle.

In contrast to cell survival, ROS produced by PRODH can also trigger cell death signals that lead to cell cycle arrest and apoptosis. For example, PRODH expression is upregulated by the p53 tumor suppressor, leading to ROS generation and cell apoptosis [12,118]. This antitumorigenic role of PRODH is linked to activation of the cell death receptor, TRAIL, and nuclear factors of activated T-Cells (NFAT) [121]. The amount of ROS that causes signaling to switch from cell survival to cell death is called the Redox Stress signaling Threshold (RST). The ROS generated by PRODH can vary significantly depending on the tissue type and cancer, with ROS levels associated with PRODH differing by up to six-fold [14]. The RST and antioxidant capacity of the microenvironment are the factors that likely contribute to the dual functionality of PRODH in promoting cell survival or cell death. A recent study found that PRODH levels were linked to mitochondrial phosphoenolpyruvate carboxykinase (PEPCK-M). When PEPCK-M is knocked out in Hela cells cultured in high glucose and glucose exhausted media, the PRODH mRNA level increases. This was observed alongside a decrease in proline levels, which limited cell growth. Increased levels of PRODH due to the depletion of PEPCK-M was further shown to impact cellular ROS levels, with a slight increase in ROS being potentially beneficial [170]. Higher levels of ROS, however, triggers cell cycle arrest and cell death through NFAT, TRAIL, and the suppression of MEK/ERK signaling pathways [121,171].

While the precise mechanisms underlying PRODH’s functional duality between pro-tumor and pro-apoptotic roles remain incompletely understood, a few hypotheses have begun to emerge. The first hypothesis centers on the complex interplay between PRODH-generated reactive oxygen species (ROS) and cellular antioxidant systems. During apoptotic signaling, cells upregulate PRODH expression to generate the ROS necessary for programmed cell death. Expression of manganese superoxide dismutase (MnSOD or SOD2), was shown to effectively counteract PRODH-derived ROS production release of cytochrome c from the mitochondria [120]. This finding showed that superoxide anion (O_2_^•−^) is the primary ROS generated by PRODH. Potential metabolic cross-talk suggests that PRODH’s functional outcome may be determined not solely by its intrinsic enzymatic activity, but rather by the balance between pro-oxidant generation and antioxidant capacity, such as SOD2 expression levels within the cellular environment.

The second hypothesis emphasizes the critical role of the cellular microenvironment in dictating PRODH function. Under hypoxic conditions, PRODH expression tends to enhance cell survival by promoting autophagy, thereby providing an adaptive mechanism for nutrient stress [109]. Conversely, normoxic conditions often favor PRODH-mediated apoptotic pathways, primarily through sustained ROS accumulation that triggers cell death cascades [119]. This oxygen-dependent functional switching indicates that environmental oxygen availability serves as a key determinant of PRODH’s cellular role, with hypoxic stress redirecting PRODH function toward cytoprotective pathways rather than cell death.

The third hypothesis proposes that protein–protein interactions modulate PRODH function through allosteric or competitive mechanisms. Evidence supporting this model includes the proposed role of estrogen receptor (ERα) interactions with p53 that diminish PRODH expression and subsequent ROS formation resulting in an anti-apoptotic effect in breast cancer cells [172,173]. Additionally, PRODH’s subcellular localization, particularly its association with mitochondrial membranes, may represent another regulatory mechanism. The enzyme’s capacity to associate with or dissociate from membrane structures remains poorly characterized, yet this dynamic localization could fundamentally alter its access to substrates, cofactors, and regulatory proteins. Such membrane-dependent functional switching would provide an additional layer of spatial and temporal control over PRODH activity, potentially explaining its context-dependent cellular effects toward ATP production versus ROS generation.

### 4.3. PYCR and Hypoxia

Due to high proliferation and insufficient vascularization, tumor tissue often faces some form of hypoxic condition. Under a hypoxic environment, reducing equivalents are not properly utilized by the ETC pathway and the NADH/NAD^+^ ratio increases. PYCR1 plays an essential role in the maintenance of the viability of tumor tissue under hypoxic conditions. PYCR1 utilizes the excess NADH-reducing equivalents caused by hypoxic conditions to reduce P5C to proline, which then is secreted out of the cell [21]. Knockout of PYCR1 affects the NAD^+^/NADH pool but not the NADP^+^/NADPH pool. Interestingly, lactate synthesis and efflux are increased in PYCR1 depleted cells under normoxia, but no change is observed under severe hypoxia. PYCR1 deficient cells also show a decrease in glutamate incorporation into TCA cycle metabolites, succinate and malate, which is further diminished under hypoxia. These findings were replicated in PYCR1-deficient 3D spheroid cells which had less volume and glutamate incorporation into TCA cycle metabolites. Overall PYCR1 supplies NAD^+^ to the TCA cycle in hypoxic conditions, thereby promoting tumorigenesis [21].

In multiple myeloma, both mRNA and protein levels of PYCR1 and PYCR2 were shown to increase in the hypoxic environment. High expressions of PYCR1 and PYCR2 are correlated with poor prognosis of multiple myeloma. Inhibition of PYCR1 leads to the suppression of multiple myeloma by increased apoptosis, and reduced proliferation. A phospho-proteome profiling of PYCR1 knockdown cells showed reduction in p-STAT1, p-p70, p-PRAS40, p-lck, p-lyn, p-msk1/2, p-yes, p-p53, p-PYK2, and p-STAT6. As p-PRAS40 controls cellular protein synthesis, the downregulation of p-PRAS40 in PYCR1-depleted cells ultimately results in the suppression of cellular protein translation. Combination therapy of multiple myeloma with bortezomib (a proteasome inhibitor) along with PYCR1 inhibition by pargyline significantly increased cell apoptosis (~3 fold) [87,174]. Although the off-target effects of pargyline cannot be ruled out.

A new role was discovered for PYCR1 in the hypoxic conditions of cancer cells that involves nuclear localization of the enzyme. IGF1R signaling was found to be upregulated in cancer cells leading to increased expression of PYCR1 through c-MYC and increased proline levels that support cancer growth [68]. Nuclear accumulation of IGF1R and PYCR1 were detected in colorectal cancer, where PYCR1 interacts with IGF1R. IGF1R phosphorylates PYCR1 at Tyr135 residue, which promotes PYCR1 interaction with nuclear transcription factor ELK4. Under hypoxia conditions, the IGF1R-PYCR1-ELK4 axis represses gene transcription through the SIRT7-mediated deacetylation of histone H3K18Ac, thereby supporting tumor growth. SIRT7 activity is dependent on NAD^+^, which increases under hypoxia conditions through PYCR1 activity [57].

## 5. Collagen, Proline Biosynthesis, and Cell Proliferation

### 5.1. Collagen Synthesis, Degradation, and Interaction in the Tumor Niche

Collagen, a key component of the extra cellular matrix (ECM), is one of the most abundant proteins in the body. Approximately 25% of collagen residues are composed of proline/hydroxyproline [175]. The long filamentous physical characteristics of collagen arises from collagens’ quaternary structure. This structure consists of three parallel polypeptide strands, called tropocollagens, that follow a repeating G-X-Y pattern. Glycine is denoted as G, while the X position is often occupied by proline, and the Y position typically filled by hydroxyproline [176]. Proline can be incorporated into both the X and the Y position of collagen. Once incorporated, proline can undergo hydroxylation as a PTM at the C4 position of collagen, facilitated by the presence of oxygen or ROS. Hydroxyproline can also be synthesized in the endoplasmic reticulum utilizing prolyl-4-hydroxlase (P4H) or prolyl-3-hydroxylase (P3H) when there is ascorbate, ferrous iron, α-ketoglutarate, and oxygen [177]. The hydroxylation of proline catalyzed by P4H occurs exclusively in the endoplasmic reticulum, while P3H is found in both the endoplasmic reticulum and the Golgi apparatus. P4H and P3H also play a role in the formation of collagen polypeptides by helping to arrange amino acids in the correct G-X-Y sequence. Once formed, the polypeptides are transported to the ECM, where they can mature into collagen strands.

Proline, which constitutes a significant portion of collagen polypeptides, is obtained from extracellular sources or biosynthesis. Proline is also readily recycled from proteins in a process involving extracellular protease-mediated cleavage [178]. The cleaved collagen peptides are then taken up by the cells, where they undergo lysosomal degradation [179,180]. Matrix metalloproteinases (MMPs) are the small group of proteins responsible for the extracellular cleavage of collagen (Figure 1B). There are 28 different MMP’s, each with a distinct function. In short, collagen degradation begins with collagenases, a subclass of MMP, which unwind the tightly wound collagen tropocollagens, producing ¼ and ¾ telopeptide fragments. The ¾ telopeptide fragments are then further processed by membrane-type, gelatinase, stromelysin, and matrilysin proteases (subclasses of MMP’s), producing short peptides 3–10 amino acids long. Prolidase (also known as PEPD) and prolinase are enzymes capable of cleaving imidodipeptideds, produced from MMPs, with C terminal proline or hydroxyproline to generate free proline or hydroxyproline molecules [181,182]. Once freed, the proline molecules can be transported to the mitochondria for proline cycling, incorporated into new proteins, and serve a wide range of other functions required by the cell.

Collagen provides a source of proline, and cancer cells have been found to regulate collagen degrading enzymes. An example of this correlation is the link between PEPD function and cancer phenotype and invasiveness. Higher activity of PEPD was observed in patients with gastric cancer, breast cancer, and endometrial cancer [183,184,185,186]. The metabolism of collagen is regulated by various factors and signals. One of these signals is transforming growth factor beta (TGFB), which can upregulate collagen production and proline anabolism [134,187]. The redox microenvironment may also impact collagen synthesis, with HIF1α potentially activating prolyl-4-hydroxylase subunit alpha 1 (P4HA1). This protein is responsible for the maturation of collagen that can assist in cancer cell migration and invasion [188]. Interestingly, when oxygen levels are decreased, creating a hypoxic environment for cells, the hydroxylation of collagen is upregulated. The source for the observed oxidation must be an alternative to oxygen, potentially in the form of ROS, ascorbate, ferrous iron, and α-ketoglutarate. The state of the endoplasmic reticulum may also impact the ability of P4H and P3H to perform hydroxylation. The microenvironment can additionally lead to the formation of hydroxyproline through the action of free ROS radicals. Collagen is directly connected to proline metabolism and plays a large role in the microenvironment makeup but is not the sole contributor to proline metabolism signaling.

Other sources of proline, aside from collagen, can originate from an individual’s diet or nutritional intake. Proline has been extensively studied in the context of wound healing with further exploration into prolines supplementation to aid the process [13,189,190,191]. Given that proline levels are important mediators of ROS and serve as a substrate for PRODH, it would be interesting to study the effect of dietary proline on cancer’s ability of growth, proliferation, and metastasis. Nearly all enzymes of proline metabolism have the potential to be a viable cancer therapeutic.

### 5.2. Regulation of Proline Biosynthesis in Different Cancers

While both PRODH and PYCRs are regulated by oncogenes, the cancer microenvironment, and available proline levels, PYCRs have been correlated to a host of regulatory pathways in various cancer contexts. These pathways are wide-ranging across several cancer subtypes with reported positive and negative effects on cancer growth and progression. PYCRs are influenced by a host of cancer-associated signals, such as EP300 in cancer-associated fibroblast (CAF), Lon protease, GCN2, c-MYC, ATP, TGFB, and Kindlin-2 [54,65,85,86,113,138,192,193,194,195,196]. Like PRODH, PYCRs can also partake in regulating metabolic signals such as SAPK/JNK, AKT, STAT3, GCN2, and mTOR, as well as contribute to ROS regulation [63,102,197]. Both the catabolism and anabolism of proline are widely regulated and used for signaling, highlighting the dual functionality of proline metabolism in maintaining cellular homeostasis. PYCR2 was found to create a positive feedback loop with AlkB homolog 5 RNA demethylase allowing for increased proliferation, migration, and invasion [103]. A study on mTORC activation-mediated transcription showed upregulation of PYCR1 and PYCR2 expression upon mTORC activation, which reverts upon ATF4 knockout (an effector of mTORC1). This finding suggests mTORC1-ATF4-mediated regulation of PYCR1 and PYCR2 [197].

PINCH-1 is part of the cell extracellular matrix, and its expression is promoted by TGF-β1 signaling [198]. It regulates proline biosynthesis by promoting the expression of both PYCR1 and P5CS. Interestingly, PINCH-1 regulates P5CS at the protein level and also physically interacts with it through its LIM domain. A LIM domain is a zinc double finger region and PINCH-1 has five of these domains. In lung adenocarcinoma and lung fibroblasts, the knockdown or inactivation of PINCH-1 leads to a corresponding decrease in P5CS expression level and a decrease in PRODH. The physical interaction between PINCH-1 and P5CS prevents leupeptin-sensitive protease-mediated degradation of P5CS [133].

Recombinant human TNF related apoptosis-inducing ligand protein (TRAIL) has been shown as an effective tumor suppressor. In non-small lung cancer cell line H1299, You et al. found that knockdown of PYCR1 increases sensitivity towards TRAIL, partially mediated by increased localization of death receptors on the cell surface [88]. Levels of PYCR were found to be upregulated in gastric cancer patient tissue samples. Increased levels of PYCR were associated with an increase in apoptosis marker cleaved caspase-3, and metastasis-associated marker proteins such as E-cadherin, p-AKT, p-PI3K, and Snail [89].

The reduced expression of mitochondrial pyruvate carrier protein (MPC1) is a frequent event in ovarian cancer and leads to reduced import of pyruvate into the mitochondria. This increases the dependency of cancer cells on glutamine as a source of acetyl-CoA. Farrok et al. showed an increase in the levels of PYCR protein upon MPC1 depletion, which supports ovarian cancer cells’ growth, ROS production, colony formation, and collagen synthesis (Type I and VI) [199]. A decreased expression of basic helix-loop-helix family member e41 (BHELH41) is correlated with a poor prognosis of bladder cancer. The overexpression of BHELH41 suppresses tumor progression. Interestingly, BHELH41 physically interacts with PYCR1 and decreases its stability through ubiquitin-mediated proteasomal degradation [90]. Increased expression of PYCR2 correlates with a poor prognosis of colorectal cancer (CRC), therefore inhibition of PYCR2 inhibits CRC tumor growth. Mechanistically, PYCR2 promotes cancer stem cell population by regulating the MASTL/Wnt/b-catenin axis to support CRC tumor growth [101]. Similarly, PYCR1 was found to be responsible for cancer stem cell maintenance of breast cancer. Cui et al. reported that the increased expression of PYCR1 in triple negative breast cancer cells as well as in spheroids leads to increased proline synthesis. Increased proline levels activate cGMP-PGK signaling to support breast cancer stem cells [69]. Zhang et al. reported an increased expression of PYCR1 in lung adenocarcinoma which enhances STAT3 phosphorylation. The activation of the JAK/STAT signaling pathway increases the expression of immunosuppressive PD-L1 to inhibit the tumors’ immune suppression and promote tumor progression. Interestingly, the inhibition of the PRODH enzyme in lung adenocarcinoma effectively attenuates PYCR1-mediated effects by inhibiting the glutamine levels [58]. In prostate cancer cells, proline and PYCR1 were found to promote the cancer stem cell-like properties though induction of the JAK-STAT signaling pathway [200].

PYCR1 is not only over-expressed in breast cancer cells but also highly expressed in CAFs. Increased expression of PYCR1 in CAFs leads to increased proline synthesis, which is then utilized for collagen synthesis and extracellular matrix support for growing cancer cells. The knockdown of PYCR1 in cancer cells moderately reduces the proliferation, while targeting ALDH18A1 (also known as P5CS) had a greater effect on limiting proline and collagen production [54]. In an attempt to identify the role of CAFs in chemoresistance and cancer progression, Fan et al. identified a senescence-like tetraspanin-8 (TSPAN8)+ myofibroblastic CAF (myCAF) subpopulation. TSPAN8+ MyCAFs confer chemoresistance to nearby breast cancer cells by promoting their stemness through secreting senescence-associated secretory phenotype (SASP) related factors. Interestingly, TSPAN8-mediated downregulation of SIRT6 increases the expression of GLS1 and PYCR1, whose secretion provides aspartate and proline to the breast cancer cells for proliferation [201]. Similarly, increased expression of PYCR1 was found in rat glioma and associated CAFs to support proliferation and angiogenesis [59]. Sawika et al. also showed an increase in PYCR1 protein, proline amino acid and decrease in PRODH protein levels in patients’ glioma cells [91]. Furthermore, targeting of pancreatic cancer-associated CAFs by extra-cellular vesicles loaded with miR-138-5p and the anti-fibrotic agent pirfenidone suppresses the pro-tumorigenic effects of CAFs [97].

A recent study explored how proline biosynthesis is utilized in certain cancer subtypes by specifically investigating the upregulation of PYCR3 in triple negative breast cancer (TNBC) cells [105]. The authors found that upregulation of PYCR3 correlates with a decrease in patient survival. TNBC cells with a knockout of PYCR3 show a decrease in cell viability and proliferation. One interesting observation of this study was the sub-localization of PYCR3. PYCR3, which is traditionally thought to be localized in the cytosol, was found to be present in the mitochondria. The authors proposed that mitochondrially localized PYCR3 may regulate mitochondrial gene expression and respiratory function. They came to these conclusions by observing that the KO of PYCR3 led to a decrease in OCR, ATP, and ETC complex levels. This was accompanied by a decrease in mitochondrial DNA and RNA levels but no change in proline levels. Furthermore, doxorubicin-resistant TNBC cells show restored sensitivity upon PYCR3 knockout [105]. While these findings are interesting, further confirmation is needed for PYCR3 sublocalization by using different antibodies and by expressing tagged version of proteins, as PYCR antibodies are known to frequently cross react with PCYR isoforms.

## 6. Therapeutic Opportunities Targeting Proline Metabolism

### 6.1. Proline Enzyme Variants and Cancer

Mutations in proline metabolic enzymes have been linked to conditions such as autosomal recessive cutis laxa type I and II (PYCR1 gene MIM 179035), hypomyelinating leukodystrophy 10 (PYCR2 gene OMIM 616420) gerodermia osteodyplastica (PYCR1 gene MIM231070), and velocardiofacial syndrome (PRODH) [202,203]. In the context of cancer, there is a knowledge gap concerning the role of proline metabolic enzyme variants in regard to cancer causation and progression. This provides an unexplored opportunity for genome-wide association screening, DNA profiling, and cancer patient proteomics to identify novel variants. While The Cancer Genome Atlas Project (TCGA) reveals mutations in proline enzymes in cancer patients, there is no evidence that mutations that impact enzyme activity have a direct role in cancer progression. For example, a recent study identified PYCR1 variant Thr171Met in patients with malignant melanoma and lung adenocarcinoma. The PYCR1 T171M variant exhibited a significant decrease in enzyme activity relative to wild-type enzyme. The decrease in enzyme activity of the variant was caused by the Met residue directly interfering with the P5C substrate binding in the active site. Because the diminished enzyme activity of the PYCR1 T171M variant is not expected to promote cancer progression, an indirect mechanism is more likely, potentially involving changes in interactions with other proteins that influence cell signaling pathways [204]. While there has been considerable research on individual proline metabolism enzymes, future studies are needed to explore linkages of mutations in proline metabolic enzymes with cancer patient outcomes, which could reveal new avenues for therapeutic interventions.

### 6.2. Inhibition of Proline Metabolic Enzymes

Developing inhibitors to target key enzymes of proline metabolism could offer effective treatment across a wide range of cancer contexts. Enzymes that show susceptibility to inhibition by biologically available molecules can be further explored by screening compound libraries of similar molecules. This approach aids in the identification of specific inhibitors with potential therapeutic applications. Physiological inhibition includes PRODH by lactate, PYCR’s by ATP, PYCR2 proline feedback inhibition, and feedback inhibition of the P5CS short isoform by ornithine [16,145,146,149]. Additionally, 4S-hydroxyproline (cis-hydroxyproline) was reported to inhibit the biosynthesis of collagen [205]. These biologically available molecules have numerous reported analogs that could be tested in cell models to identify more potent inhibitors [36]. Recent reviews exploring the potential uses of proline analogs, along with historical drugs, demonstrate the broad potential of proline metabolism-focused cancer therapeutics [36].

**Figure 3:**
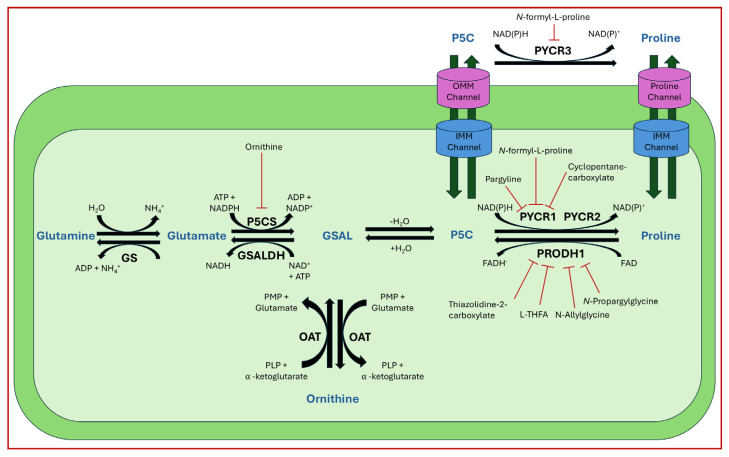
Known drug targets of proline metabolism. The core proline metabolic pathway is shown above with selected compounds targeting PYCR1 and PRODH1.

Steps toward developing small molecule inhibitors against proline metabolic enzymes have largely focused on PRODH and PYCR1 (Figure 3). Table 2 summarizes the different types of inhibitor compounds that have been reported thus far. Non-covalent reversible and covalent inhibitors of PRODH have been kinetically and structurally characterized and used in cell cultures and animal studies with successful outcomes [206]. *S*-(-)-tetrahydro-2-furoic acid (THFA) was first reported as a reversible inhibitor of the PRODH domain in the bacterial proline utilization, A protein, which combines PRODH and GSALDH activity on a single polypeptide [147,207]. THFA has subsequently been shown to inhibit spheroidal growth of breast cancer cells and in mouse models of breast cancer to diminish metastasis to the lung [126].

A promising mechanism-based inhibitor against PRODH, *N*-propargylglycine (NPPG), was originally characterized using bacterial PRODH and PutA enzymes [211,216]. The Tanner group also recently reported new mechanism-based inhibitors, *N*-allylglycine and but-3-yn-2-ylglycine (B32G) [206,214]. The inactivation mechanisms for NPPG and B32G start with oxidation of the compound by the enzyme and the formation of reduced FAD. Nucleophilic attack of the oxidized intermediate by the reduced FAD N5 then leads to a covalent adduct of the compound between the FAD N5 and ε-nitrogen of an active-site lysine conserved in PRODH enzymes. Another class of covalent inhibitors of PRODH includes S-heterocycles 1,3-dithiolane-2-carboxylate, and thiazolidine-2-carboxylate [210,215]. These novel compounds also form adducts with the N5 of the FAD but through different mechanisms, such as a blue light activated mechanism in the case of 1,3-dithiolane-2-carboxylate [215].

The effectiveness of covalent inhibitors as chemical probes of cancer metabolism has been demonstrated in cell culture and animal studies. The mechanism-based inhibitors were shown to decrease protein levels of PRODH1 and PRODH2 in breast and liver cancer cell lines, indicating covalent inactivation results in cellular degradation of these enzymes [214]. C57BL/6J mice gavaged for five days with NPPG and B32G resulted in a significant decrease in PRODH1 and PRODH2 levels in the liver [214]. Breast tumor xenograft mouse models also showed significant loss of PRODH1 and oral treatments with NPPG resulted in decreased PRODH1 in the brain, indicating a potential treatment strategy for brain cancer [129,217]. The observation that NPPG also targets PRODH2 has been shown to be a potential therapeutic strategy for the treatment of hyperoxaluria [218].

The best characterized compound targeting PYCR1 thus far is *N*-formyl-L-proline (NFLP) [208]. NFLP is a competitive inhibitor of PYCR1 (*K*_I_ = 100 µM) and in breast cancer cells, blocks proline biosynthesis and spheroid growth [208]. Recent compound screening aimed at discovering molecules that are more selective for PYCR1 relative to other PYCR isoforms, identified (S)-tetrahydro-2H-pyran-2-carboxylic acid as a selective inhibitor (*K*_I_ = 70 µM) [209]. This compound is 30-fold more selective for PYCR1 than PYCR3 (*K*_I_ = 2100 µM) [209]. Whether (S)-tetrahydro-2H-pyran-2-carboxylic acid is effective at inhibiting proline biosynthesis in cells has not been reported. Inhibitors that specifically target PYCR isoforms present a valuable opportunity to selectively disrupt the proline cycle, potentially hindering proline metabolism and affecting cancer cell growth.

Identifying new compounds against proline metabolic enzymes for strategic use as chemical probes for cancer research will require continued significant effort. Key insights into structure–affinity relationships have been gained through compound screening studies of PRODH and PYCR1 by the Tanner group [144,209]. The similar size of proline and P5C make is challenging to design non-covalent reversible compounds that are selective for PRODH and PYCR. More studies that explore the structure–affinity relationships of these enzymes are needed to better define the chemical space available for new chemical probes and lead compounds. The mechanism-based inhibitors have the advantage of high selectivity for PRODH enzymes relative to PYCR isoforms and show promise for further optimization. The amount of selectivity required between PRODH and PYCR will be highly influenced by the type of cancer and mechanism of action. For example, the consequence of PRODH1 being inhibited off-target in healthy tissues when PYCR1 is the primary target in cancer cells will need to be considered. A promising result, however, was achieved with mice given therapeutic treatment with L-THFA, a reversible inhibitor of both PRODH1 and PYCR1 (see Table 2). Mice showed no adverse impact on healthy tissue when treated with L-THFA while lung metastases was effectively blocked [126]. Thus, potential dual inhibition of PRODH1 and PYCR1 in healthy tissue did not have a negative consequence on the therapeutic outcome. Even so, undesired metabolic effects of compounds that potentially inhibit PRODH1 and PCYRs will need to be fully assessed in future clinical studies.

While several compounds have already been identified, there is an ongoing effort to screen and discover new compounds to target proline metabolism [144]. Many of the compounds targeting proline machinery have been tested in vitro, leaving room for further studies in vivo. These experiments would clarify the effectiveness of these compounds as potential drugs and cancer therapeutics. There is also the potential to develop a new class of inhibitors by exploiting amino acid residues outside the immediate active site regions. This could be achieved by building molecules that specifically modify residues near the active site or dimerization domains such as in PYCRs.

### 6.3. Alternative Targets of Proline Metabolism

Beyond core enzymes of proline metabolism, adjacent pathways such as collagen recycling and ornithine and glutamine metabolism, present additional therapeutic targets. Collagen, in particular, has been shown to assist in cancer metastasis and serve as an additional reserve of proline and hydroxyproline [176,188,219]. A potential anticancer therapeutic strategy would be to impair collagen synthesis or maturation. 4-S-hydroxyproline has been examined in the context of pancreatic carcinoma, showing an inhibition of tumor growth. However, the same compound has demonstrated confounding results when applied to rat mammary carcinoma cells [205,220,221]. Further experiments are needed to fully clarify the effects of this compound on collagen maturation.

Other reports examining collagen in the context of cancer found that collagen maturation is important for metastatic progression and induced by TGFB [192,222]. In studies where collagen is upregulated, it would be interesting to examine proline metabolism. Since proline makes up a significant portion of collagen, some cancer cell subtypes that rely on collagen formation may be sensitive to inhibition of PYCR’s, proly-4-hydroxylases, and steroisomers of proline that inhibit protein synthesis [175]. Another avenue involves targeting the ornithine and glutamine pathways. P5C/GSAL derived from ornithine is primarily utilized by PYCR3 and is closely linked to the urea pathway. By inhibiting ornithine aminotransferase and depleting ornithine levels, the capacity for proline-dependent respiration in the mitochondrion would be expected to decrease. PYCR3 metabolism may be the most complex of the PYCR isoforms due to its preferred substrate, GSAL derived from ornithine. The reliance of PYCR3 on ornithine metabolism makes it heavily dependent on P5C/GSAL mitochondrial transport.

Targeting respiratory complexes in the mitochondrion may provide alternative strategies for inhibiting proline catabolism. The inhibition of complex V by oligomycin was found to decrease proline levels in whole cell lysates of HeLa cells and the inhibition of complex I by piericidin decreases proline levels in the mitochondrial matrix. Pyruvate supplementation in complex V inhibited conditions (reported earlier to regenerate NAD under respiratory complex dysfunction) further decreased proline levels in whole cell lysates [223]. The inhibition of complexes I and V can cause the cell to preserve the mitochondrial membrane potential and pyrimidine synthesis due to the raspatory chain being disrupted. These key processes require aspartate to stabilize the matrix energy pathways. The influx of aspartate leads to the transfer of glutamate being shipped out of the mitochondrial matrix to promote an influx of aspartate. With glutamate being a substrate for proline synthesis, this could be one reason that proline levels were observed to decrease [223]. More studies on inhibiting respiratory complexes need to be conducted to better understand the impact on proline levels.

## 7. Conclusions

Proline metabolism has multiple effects on cancer progression that will require in depth studies on different types of cancers to fully reveal the mechanisms most relevant for therapeutics. Currently, two leading targets for therapeutic compound development are PRODH1 and PYCR1 due to their necessity in a broad number of cancer cells for hyperproliferation and advancement toward metastasis. This review has compiled the current information of proline metabolism’s regulation in cancer cells, gene expression changes, mutations, and proline-dependent pathways such as collagen synthesis.

## Figures and Tables

**Table 1:** Proline metabolic enzyme expression levels and effects on cancer progression.

Proline Enzyme	Expression Level	Cancer Progression	References
PYCR1	UP	PRO	[16,21,54,55,56,57,58,59,60,61,62,63,64,65,66,67,68,69,70,71,72,73,74,75,76,77,78,79,80,81,82,83,84,85,86,87,88,89,90,91,92,93,94]
	DOWN	PRO	[95,96]
	DOWN	ANTI	[97]
PYCR2	UP	PRO	[92,93,94]
	UP	ANTI	[98,99,100,101,102,103]
PYCR3	DOWN	ANTI	[67,103,104]
	UP	PRO	[105,106]
PRODH1	UP	PRO	[51,52,67,91,95,107,108,109,110,111,112,113,114,115,116]
	UP	ANTI	[117,118,119,120,121,122,123,124,125]
	DOWN	ANTI	[125,126,127,128,129,130,131,132]
PRODH2	DOWN	PRO	[11]
P5CS	UP	PRO	[11,106,133,134]
	DOWN	ANTI	[19,135,136,137,138]
P5CDH	DOWN	PRO	[66]

**Table 2:** Established inhibitors of proline metabolic enzymes.

Compound	Structure	Type of Inactivation	Target Enzyme	References
*N*-formyl-L-Proline	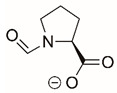	Reversible	PYCR1	[208]
		Reversible	PYCR3	[208]
Pargyline	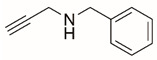	Unreported	PYCR1	[174]
Cyclopentanecarboxylate	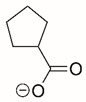	Reversible	PYCR1	[208]
L-thiazolidine-4-carboxylate	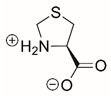	Reversible	PYCR1	[208]
*N*-(4-bromobenzyl)-*N*-methylprop-2-yn-1-amine	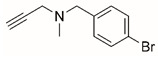	Unreported	PYCR1	[174]
L-tetrahydro-2H-pyran-2-carboxylic acid	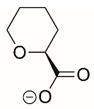	Reversible	PYCR3	[209]
		Reversible	PYCR1	[209]
ATP	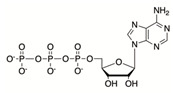	Reversible	PYCR1-3	[145]
Proline	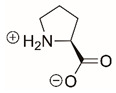	Reversible	PYCR1	[208]
L-thiazolidine-2-carboxylate	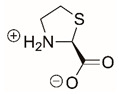	Reversible	PYCR1	[208]
		Covalent	PRODH	[210]
L-tetrahydro-2-furoic acid	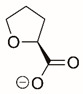	Reversible	PYCR1	[208]
		Reversible	PRODH1	[147]
*N*-allylglycine	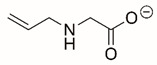	Covalent	PRODH	[206]
2-prop-2-enylsulfanylacetate	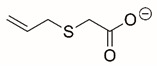	Reversible	PRODH	[206]
*N*-propargylglycine	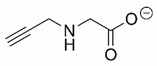	Covalent	PRODH	[206,211]
2-prop-2-ynylsulfanylacetate	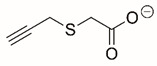	Reversible	PRODH	[206]
Cyanomethyl sulfanylacetate	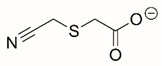	Reversible	PRODH	[206]
S-5-oxo-2-tetrahydrofurancarboxylic acid	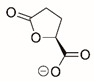	Reversible	PRODH1	[129]
4-methylene-L-proline	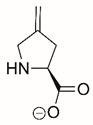	Covalent	PRODH1	[211,212]
L-lactate	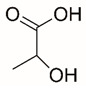	Reversible	PRODH1	[147,213]
(S)-but-3-yn-2-ylglycine	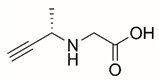	Covalent	PRODH	[214]
1,3-dithiolane-2-carboxylate	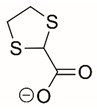	Reversible and Covalent (photoinduced)	PRODH	[215]
Tetrahydrothiophene-2-carboxylate	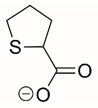	Reversible and Covalent (photoinduced)	PRODH	[215]
Ornithine	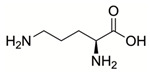	Reversible	P5CS	[149]

## Abbreviations

The following abbreviations are used in this manuscript:

PRODH1	Proline dehydrogenase isoform 1
PRODH2	Proline dehydrogenase isoform 2
NEAA	Non-essential amino acid
GSAL	L-glutamate-γ-semialdehyde
P5C	L-Δ^1^-pyrroline-5-carboxylate
P5CS	L-Δ^1^-pyrroline-5-carboxylate synthase
PYCR	L-Δ^1^-pyrroline-5-carboxylate reductase
PLP	Pyridoxal-5-phosphate
OAT	Ornithine-γ-aminotransferase
PRODH	Proline dehydrogenase
FAD	Flavin adenine dinucleotide
P5CDH	L-Δ^1^-pyrroline-5-carboxylate dehydrogenase
SLC6A19	Solute carrier family 6 member 19
SLC6A7	Solute carrier family 6 member 7
PTM	Post translational modifications
GABA	Gamma-aminobutyric acid
GAD	Gamma-aminobutyric acid receptors
PIG6	p53 inducible gene
ChIP	Chromatin immunoprecipitation assay
LDL	Low-density lipoprotein
oxLDL	oxidized low-density lipoprotein
KSHV	Kaposi’s sarcoma-associated herpesvirus
L-THFA	L-tetra-hydro-2-furoic acid
CBP	CREB binding protein
USP9x	Ubiquitin-specific peptidase 9 X-linked
UCP	Uncoupling proteins
VHL	Von-Hippel Lindau ubiquitin ligase
NFAT	Nuclear factors of activated T-Cells
RST	Redox stress signaling threshold
PEPCK-M	Mitochondrial phosphoenolpyruvate carboxykinase
ECM	Extra cellular matrix
P4H	Prolyl-4-hydroxlase
P3H	Prolyl-3-hydroxylase
MMP	Matrix metalloproteinases
TGFB	Transforming growth factor beta
P4HA1	Prolyl 4 hydroxylase subunit alpha 1
CAF	Cancer-associated fibroblast
TRAIL	TNF related apoptosis inducing ligand protein
MPC1	Mitochondrial pyruvate carrier protein
BHELH41	Basic helix-loop-helix family member e41
CRC	Colorectal cancer
myCAF	myofibroblastic cancer-associated fibroblast
SASP	Senescence-associated secretory phenotype
TNBC	Triple negative breast cancer cells
THFA	*S*-(-)-tetrahydro-2-furoic acid
B32G	But-3-yn-2-ylglycine
NFLP	*N*-formyl-L-proline
